# Knotting of Guide Wires During Percutaneous Nephrolithotomy: A Complex Intraoperative Challenge

**DOI:** 10.7759/cureus.92972

**Published:** 2025-09-22

**Authors:** Ashay A Patil, Shashank Sharma, Amol Kamble

**Affiliations:** 1 Urology, Grant Government Medical College and Sir J.J. Group of Hospitals, Mumbai, IND

**Keywords:** double-j stent, endourology, fluoroscopic guidance, guidewire knotting, intraoperative complication, minimally invasive urology, percutaneous nephrolithotomy, renal calculi, stone management, surgical troubleshooting

## Abstract

Percutaneous nephrolithotomy (PCNL) is the gold standard for managing complex renal calculi, offering high stone-free rates with minimal invasiveness. Despite its safety, intraoperative complications can present significant challenges. This case highlights a rare but critical complication - guidewire knotting during PCNL - and outlines the intraoperative strategy employed for its successful resolution.

A 35-year-old male with recurrent left flank pain and prior retrograde intrarenal surgery (RIRS) and PCNL underwent prone PCNL for residual renal calculi and a retained double-J (DJ) stent. During renal access, significant resistance was encountered while advancing a super-stiff guidewire. Fluoroscopy confirmed guidewire knotting within the pelvicalyceal system, posing a risk of ureteral injury or fracture. Gentle traction failed, necessitating a secondary puncture to establish an alternative access route, enabling safe removal of the knotted guidewire. The procedure continued successfully, achieving complete stone clearance, and a new DJ stent was placed.

The patient had an uneventful recovery, with stone-free status confirmed at six weeks. Guidewire knotting is a rare but significant challenge in PCNL, particularly in patients with prior interventions. This case underscores the importance of meticulous fluoroscopic monitoring, early recognition of intraoperative resistance, and a structured problem-solving approach. Awareness of such complications and alternative access strategies can help optimize patient safety and surgical outcomes in complex stone disease.

## Introduction

Percutaneous nephrolithotomy (PCNL) is the gold standard treatment for large (>2 cm), staghorn, or complex renal calculi, as endorsed by the European Association of Urology (EAU) and American Urological Association (AUA) guidelines [1,2]. The procedure involves establishing a percutaneous tract into the renal collecting system, followed by tract dilatation over a guidewire to allow nephroscope insertion and stone removal [3]. Achieving secure access with a properly placed guidewire is crucial to procedural success and patient safety, as loss of access may require re-puncture, increasing risks such as hemorrhage, adjacent organ injury, or infection [4].

Reported PCNL complication rates range from 15-25%, with major complications occurring in approximately 5-10% of cases [5]. Guidewire-related issues, including kinking, misplacement, or knotting, are exceedingly rare but can significantly complicate tract dilation and increase operative risk [6,7]. Knotting of guidewires during PCNL has been described in only a few case reports and case series, including the recent series by Para et al., which outlined successful percutaneous retrieval strategies for knotted or stripped guidewires [8].

Understanding this rare technical complication is important for urologists, especially trainees or generalists who may not routinely perform complex endourologic procedures. Here, we present a case of intraoperative guidewire knotting during PCNL in a patient with prior renal surgery, describe the mechanism and clinical implications, and outline a stepwise approach to safe resolution.

## Case presentation

A 35-year-old male with a history of recurrent left renal calculi and multiple prior urological interventions, including retrograde intrarenal surgery (RIRS) and a prior PCNL, presented with persistent left flank pain. He had undergone RIRS 18 months prior, followed by PCNL 12 months prior for residual stones. A double-J (DJ) stent had been placed at the end of the first PCNL but was inadvertently retained. He presented to our center six months after the last PCNL, at which point imaging revealed the encrusted retained stent with residual upper calyceal calculi.

The decision was made to proceed with PCNL for stone clearance and stent removal. The procedure was performed under general anesthesia in the prone position. A lower calyceal puncture was achieved under fluoroscopic guidance, and a super-stiff Amplatz guidewire was advanced. However, resistance was noted during sequential fascial dilation over the wire. Fluoroscopy revealed abnormal coiling of the guidewire within the collecting system. Subsequent manipulation and attempts to withdraw the dilator were met with resistance.

Intraoperative fluoroscopy demonstrated a knot in the guidewire, with its distal end anchored within the upper pole. Recognizing the risk of ureteral trauma and wire fracture, the procedure was paused. An upper pole puncture was then created to gain a secondary access tract. Through this tract, stone clearance was achieved, and the knotted guidewire was successfully retrieved intact using graspers. A new DJ stent was placed, and final fluoroscopy confirmed complete stone clearance.

Throughout the procedure, the patient remained hemodynamically stable with no signs of bleeding, extravasation, or vital instability. No injury to the pelvicalyceal system was noted on postoperative imaging. The retrieved knotted guidewire was confirmed to be a super-stiff wire (0.035-inch diameter; Boston Scientific, Marlborough, MA, USA).

Retrospective review of the preoperative CT urography did not reveal gross anatomical anomalies, though a relatively capacious renal pelvis and elongated infundibulum may have contributed to intrarenal guidewire mobility.

**Figure 1:**
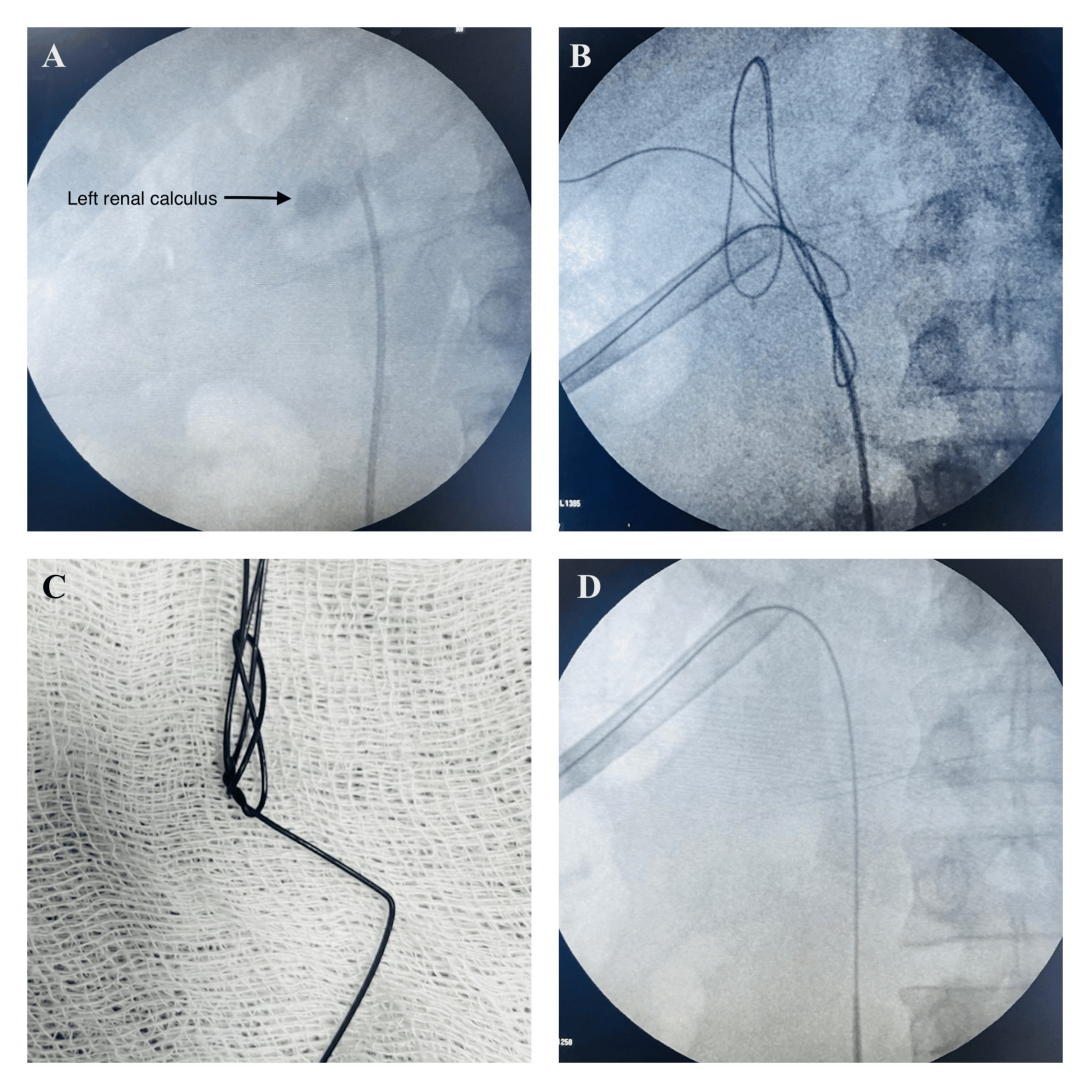
Sequential imaging demonstrating the intraoperative course and management of guidewire knotting during percutaneous nephrolithotomy (PCNL). (A) Preoperative fluoroscopy showing renal calculus and a ureteric catheter. (B) Intraoperative fluoroscopy revealing a knotted guidewire within the collecting system. (C) Removed knotted guidewire after successful retrieval. (D) Postoperative fluoroscopy confirming complete stone clearance and proper guidewire placement.

Figure 1A-1D supports the findings visually. Key clinical clues suggesting guidewire knotting included unexpected resistance during tract dilation, fluoroscopic imaging showing coiling or looping, and persistence of resistance even on attempted withdrawal.

## Discussion

Guidewire-related complications in endourology, although rare, can present significant intraoperative challenges. Among these, guidewire knotting is exceedingly uncommon, with only a few cases reported in the literature [6-8].

In the present case, resistance during guidewire manipulation and subsequent fluoroscopic imaging confirmed a knot, necessitating abandonment of that tract and creation of a second access. This aligns with previous reports where intraoperative resistance was the first clue, and fluoroscopic imaging was essential for diagnosis and safe management [8].

Some studies suggest that coiled or highly flexible hydrophilic guidewires may predispose to such complications due to their mechanical properties, including increased tendency to loop or form knots [6]. However, there is no definitive comparative data on incidence between different wire types, and no formal recommendations can be made based on current evidence [6,7].

While prior renal interventions or distorted anatomy may theoretically increase the risk of guidewire knotting, cases have also been reported in anatomically normal collecting systems [8]. Therefore, vigilance during wire manipulation is crucial regardless of patient history.

Successful management, as demonstrated in this case, relies on early recognition, gentle handling, and avoidance of forceful traction. Using real-time fluoroscopy to confirm wire position, abandoning a compromised tract, and establishing a new puncture are strategies recommended in the literature to minimize complications [8].

Finally, this case underscores the importance of effective team communication and intraoperative adaptability, principles emphasized across urological surgery and critical for safe and successful outcomes [1,2,5].

## Conclusions

This case highlights the rare occurrence of guidewire knotting during PCNL and its successful intraoperative management. Surgeons must maintain a high index of suspicion when encountering resistance during guidewire manipulation and should immediately confirm the position fluoroscopically before proceeding.

The contributing factors in this case likely included anatomical distortion from a staghorn stone, prior instrumentation, and the use of a long hydrophilic guidewire - each increasing the risk of redundancy and looping. While knot formation is rare, its consequences can be serious. As such, continuous fluoroscopic guidance, gentle handling, and avoidance of excessive guidewire advancement are essential preventive measures.

If knotting is suspected or confirmed, forceful retrieval should be avoided. Alternative strategies such as snaring, endoscopic assistance, or obtaining a new tract, as in our case, can ensure patient safety and procedural success.

Finally, this case underscores broader surgical lessons: complications can arise even in routine steps of a procedure, and a combination of vigilance, intraoperative adaptability, and clear team communication remains key to successful outcomes. Larger case series or experimental models are warranted to better understand the biomechanical and procedural factors leading to guidewire knotting during endourological procedures.
